# Anti-Yo Mediated Paraneoplastic Cerebellar Degeneration Associated with Pseudobulbar Affect in a Patient with Breast Cancer

**DOI:** 10.1155/2017/8120689

**Published:** 2017-03-09

**Authors:** Allison N. Martin, Patrick M. Dillon, David E. Jones, David R. Brenin, David A. Lapides

**Affiliations:** ^1^Department of Surgery, University of Virginia, Charlottesville, VA, USA; ^2^Department of Medicine, University of Virginia, Charlottesville, VA, USA; ^3^Department of Neurology, University of Virginia, Charlottesville, VA, USA

## Abstract

Paraneoplastic cerebellar degeneration (PCD) is a rare anti-Yo mediated paraneoplastic syndromes rarely that is infrequently associated with breast cancer. We present a case of a 52-year-old female presenting with diplopia, gait instability, dysarthria, dysphagia, nystagmus, and, most notably, new onset paroxysmal episodes of uncontrollable crying concerning for pseudobulbar affect (PBA). Serologic testing showed anti-Yo antibodies. The patient was found to have stage IIIA breast cancer as the inciting cause of the paraneoplastic syndrome. The patient was treated with neoadjuvant chemotherapy, modified radical mastectomy, adjuvant Herceptin, and pertuzumab. She was given IVIG for paraneoplastic syndrome, antidepressants, and dextromethorphan-quinidine (Nuedexta), the first FDA-approved therapy for PBA. With multimodality therapy, she demonstrated significant improvement in neurologic and mood symptoms associated with PCD and PBA.

## 1. Introduction

Paraneoplastic neurologic disorders are quite uncommon in breast cancer and are seen less frequently in breast cancer than in the high mutational burden cancer types such as lung, melanoma, and head and neck. It is postulated that random mutations in cancer cells occasionally lead to neoantigens or reexpression of embryo-fetal antigens against which the human immune system in turn responds. Unfortunately, the expression of neoepitopes and reexpression of embryo-fetal epitopes sometimes results in T cell and antibody responses against components of the nerve cell and supporting glial cells. The anti-Purkinje antibody, otherwise known as anti-Yo, has been associated with paraneoplastic cerebellar degeneration (PCD) and two other syndromes, paraneoplastic sensory peripheral neuropathy and the opsoclonus-myoclonus syndrome [1]. Anti-Yo has not previously been associated with pseudobulbar affect (PBA).

PCD associated with anti-Yo antibodies has been described in prior reports associated with various neoplasms and presents with ataxia, nystagmus, vertigo, and dysarthria [2, 3]. PBA, characterized by uncontrolled emotional outbursts, such as crying or laughter inappropriate to the social setting, in a patient with a primary breast neoplasm and anti-Yo antibodies has not been previously reported [2, 4]. PBA is classically seen in stroke, multiple sclerosis (MS), amyotrophic lateral sclerosis (ALS), and traumatic brain injury (TBI), which affect the cerebral cortex and brainstem [4, 5]. It is often incorrectly identified as depression—the diagnosis can be even more challenging in the setting of a new cancer diagnosis.

PBA is a type of affect lability characterized by sudden, involuntary, and distressing outbursts of laughing and/or crying that are often exaggerated or disconnected from mood state or social context [6–8]. PBA episodes tend to be stereotypical, can last from seconds to several minutes, and often occur multiple times per day. PBA is thought to occur as a result of injury or disease that disrupts pathways regulating emotional expression, or affect, including the corticobulbar tracts and basal ganglia.

PCD associated with anti-Yo antibodies has been described in patients with breast and ovarian cancers [2, 9]. Symptoms seen in anti-Yo PCD are a byproduct of cytotoxic T cell attack on Purkinje cells ultimately leading to pancerebellar dysfunction [10]. This class of paraneoplastic syndromes stands in contrast to others like myasthenia gravis, where antibodies to surface membrane proteins produce a direct pathological effect. Accordingly, PCD does not respond to intravenous immunoglobulin (IVIG) but requires treatment of the underlying neoplasm and symptomatic management of neurologic sequelae. Unfortunately, while early treatment typically improves mortality related to breast cancer, PCD results in significant morbidity, leaving most individuals dependent on assistance for activities of daily living.

## 2. Case Report

**Figure 1:**
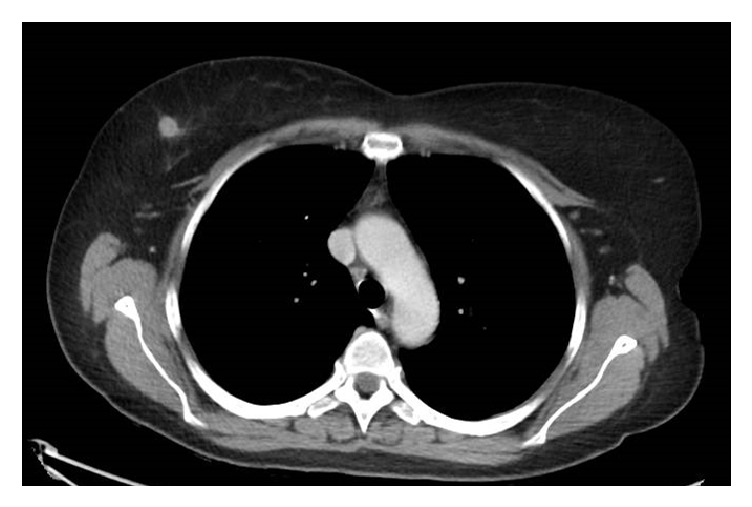
CT scan of the chest at the time of diagnosis demonstrated a round, hyperintense, 1.0 cm lesion in the upper outer quadrant of the right breast.

A 52-year-old previously healthy Caucasian female presented to the emergency department with a 1-month history of diplopia, ataxia, dysarthria, and dysphagia. Her husband reported crying spells up to 50 times per day prompted by seemingly benign occurrences. She was found to have prominent downbeat nystagmus, skew deviation, right sixth nerve palsy, cerebellar overshoot with smooth pursuits, severe cerebellar dysarthria, and profound truncal and gait ataxia. She was admitted and underwent lumbar puncture, showing RBC 1, WBC 58 (83% lymphocytes, 15% atypical lymphocytes, 2% monocytes), protein 109 mg/dL, and glucose 49 mg/dL. Magnetic resonance imaging demonstrated chronic microvascular changes in the deep white matter. A serologic paraneoplastic panel confirmed anti-Yo antibodies, 1 : 3840 (reference range < 1 : 240). A CT scan of the chest (Figure 1) to search for a lung cancer surprisingly showed a focal, right 1.5 cm breast nodule and axillary lymphadenopathy. Subsequent diagnostic mammogram revealed BI-RADS 5 finding of an irregular 1.2 cm mass with fine, pleomorphic microcalcifications. Core biopsy revealed an estrogen receptor negative/progesterone receptor negative/HER2neu positive, grade II, infiltrating ductal carcinoma with involvement of the biopsied node.

The patient received neoadjuvant nonanthracycline chemotherapy regimen with docetaxel, carboplatin, trastuzumab, and pertuzumab, with excellent clinical response. She underwent modified radical mastectomy six months following initial presentation. Surgical pathology demonstrated no residual tumor and ten lymph nodes without pathologic abnormality indicating complete pathologic response. She received postoperative chest and axillary radiation and was treated with adjuvant trastuzumab and pertuzumab. She returned to neurology clinic 45 days following initial presentation and 25 days following initiation of chemotherapy regimen but displayed persistent PCD symptoms. Intravenous immunoglobulin (IVIG) 2 g/kg was administered over three days and then monthly. The patient enjoyed periods of symptomatic improvement following each treatment. For treatment of PBA, dextromethorphan-quinidine (Nuedexta) 20-10 mg was initiated, resulting in decreased frequency in crying spells to 2-3 per day. Once she started treatment there was no further neurologic decline, but unfortunately even three years after initial presentation she still demonstrated severe truncal and limb ataxia. With extensive speech therapy, the dysarthria slightly improved.

**Figure 2:**
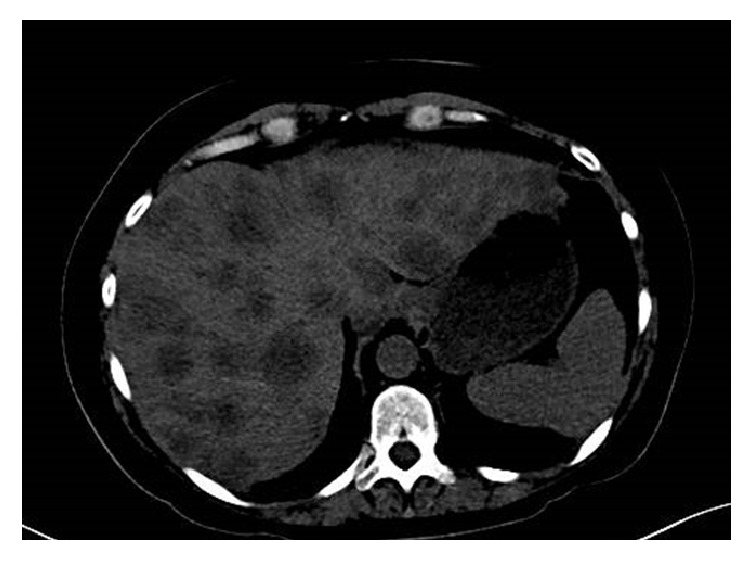
CT scan of the abdomen at the time of recurrent disease demonstrated innumerable lesions in the liver.

Unfortunately, the breast cancer recurred in the liver during year three. One of the largest liver metastases was biopsied (Figure 2), and it was morphologically similar to the original breast cancer with the same receptor pattern. The recurrent disease did not elicit exacerbation of the neurologic syndromes. The patient was treated with standard courses of lapatinib plus capecitabine and then with ado-trastuzumab. She succumbed to the disease three months after finding the metastases in the liver. She was never found to have brain metastases. A postmortem was not permitted by the family.

## 3. Discussion

This is the first report of PBA in a patient with anti-Yo mediated PCD secondary to ductal carcinoma of the breast. Since a connection between PCD and breast and gynecologic cancers is known, there is a general recommendation that patients presenting with anti-Yo PCD should undergo screening for breast cancer and gynecologic cancers. There have been suggestions that women with PCD, whose underlying cause is not apparent, might benefit from hysterectomy/oophorectomy to search for occult cancer [11–14].

PBA in this case most likely represents a rare manifestation of anti-Yo mediated PCD. PBA is most frequently seen in patients with strokes, advanced ALS, MS, and TBI; however, its pathophysiology remains poorly understood. PBA is thought to be related to decreased regulation of the motor output of emotion due to alterations in inhibitory input of serotonin and glutamate on the pons [4, 5]. This input is modulated by inhibitory signals from the somatosensory cortex and the cerebellum. Decreased inhibitory signalling leads to dysregulation and emotional lability. Notably, the cerebellum's involvement in this regulatory pathway was first described by Schmahmann and Sherman in 1998 and was originally termed “cerebellar cognitive affective syndrome” [15]. They observed among 20 patients with cerebellar lesions the occurrence of behavioural changes, executive function changes, and personality changes with either blunting of affect or disinhibition and language deficits. Parvizi et al. further described uncontrollable episodes of laughter or crying and described the cerebropontocerebellar pathways responsible for this disinhibition and refuted older views that excluded cerebellar role in execution of crying and laughter [16–18]. These groups propose that the pontocerebellar fibers may play a large role in emotional modulation [4]. McKeon et al. indicate that anti-Yo antibodies impact not only cerebellar function but also the pyramidal tract and the brainstem [19]. The PBA in this case may be related to cerebellar involvement of the anti-Yo PCD or to extracerebellar effects. Given the primary cerebellar syndrome presented here, the case supports continued investigation into cerebellar involvement as the underlying pathophysiology leading to PBA.
